# Transmission ecosystems of *Echinococcus multilocularis* in China and Central Asia

**DOI:** 10.1017/S0031182013000644

**Published:** 2013-06-05

**Authors:** PATRICK GIRAUDOUX, FRANCIS RAOUL, EVE AFONSO, ISKENDER ZIADINOV, YURONG YANG, LI LI, TIAOYING LI, JEAN-PIERRE QUÉRÉ, XIAOHUI FENG, QIAN WANG, HAO WEN, AKIRA ITO, PHILIP S. CRAIG

**Affiliations:** 1 Chrono-environment lab, UMR6249, University of Franche-Comté and CNRS, Besançon, France; 2 Institut Universitaire de France, Paris, France; 3 Epidemiology group, Vetsuisse Faculty, University of Zurich, Switzerland; 4 Ningxia Medical University, Yinchuan, Ningxia; 5 Department of Wildlife Management and Ecosystem Health, Yunnan University of Finance and Economics, China; 6 Institute of Parasitology, Sichuan Center for Disease Control, Chengdu, China; 7 UMR CBGP (INRA/IRD/Cirad/Montpellier SupAgro), INRA, Campus international de Baillarguet, CS 30016, F-34988 Montferrier-sur-Lez cedex, France; 8 Key laboratory on echinococcoses, First Affiliated Hospital of the Xinjiang Medical University, Urumqi, China; 9 Department of Parasitology, Asahikawa Medical University, Japan; 10 Cestode Zoonoses Research Group, School of Environment and Life Sciences, University of Salford, UK

**Keywords:** Ecohealth, disease transmission ecology, vole, small mammal, pika, fox, population outbreak, Kyrgyzstan, alveolar echinococcosis, landscape

## Abstract

From continental to regional scales, the zoonosis alveolar echinococcosis (AE) (caused by *Echinococcus multilocularis*) forms discrete patches of endemicity within which transmission hotspots of much larger prevalence may occur. Since the late 80s, a number of hotspots have been identified in continental Asia, mostly in China, wherein the ecology of intermediate host communities has been described. This is the case in south Gansu, at the eastern border of the Tibetan plateau, in south Ningxia, in the western Tian Shan of Xinjiang, and in the Alay valley of south Kyrgyzstan. Here we present a comparative natural history and characteristics of transmission ecosystems or ecoscapes. On this basis, regional types of transmission and their ecological characteristics have been proposed in a general framework. Combining climatic, land cover and intermediate host species distribution data, we identified and mapped 4 spatially distinct types of transmission ecosystems typified by the presence of one of the following small mammal ‘flagship’ species: *Ellobius tancrei, Ochotona curzoniae, Lasiopodomys brandtii* or *Eospalax fontanierii*. Each transmission ecosystem had its own characteristics which can serve as a reference for further in-depth research in the transmission ecology of *E. multilocularis*. This approach may be used at fine spatial scales to characterize other poorly known transmission systems of the large Eurasian endemic zone, and help in consideration of surveillance systems and interventions.

## INTRODUCTION

In the past three decades, *Echinococcus multilocularis*, the causative agent of human alveolar echinococcosis (AE), has been newly reported in countries of Europe (Davidson *et al.* 2012) and discovered in foci of relatively high incidence in China and neighbouring countries of Central Asia and Siberia (Craig *et al.* 1992; Ito *et al.* 2003, 2010; Li *et al.* 2005; Yang *et al.* 2006; Torgerson *et al.* 2009; Ziadinov *et al.* 2011; Konyaev *et al.* 2012). Although the distribution range of the parasite is described as being widespread in the northern hemisphere, large regional gaps and differences are observed. For instance, in Europe, the epidemiological status of *E. multilocularis* has changed since the 1990s with a large continental extension of its range. However, it still remains patchy with large discontinuities of occurrence within and between the traditional high endemicity areas of mid-altitude mountains of the Alpine arc, its surroundings and newly discovered foci of northern Europe (Combes *et al.* 2012; Davidson *et al.* 2012). Human AE cases in France are over-dispersed in a nested hierarchy of clusters varying in both space and time (Said Ali *et al.* – see this Special Issue of Parasitology, Vol. 140, 2013). Within a hotspot of higher endemicity, fine grain spatial distribution of the parasite and its hosts are still clustered. For instance, in a study carried out in eastern France, Raoul *et al.* (2001) found that 12% of fox definitive hosts harbour 76% of the adult parasite biomass. Furthermore, over the same extensive area, although the average prevalence in vole intermediate hosts was usually <0·01%, it could be >100x larger in some local foci (Giraudoux *et al.* 2002). Similar patterns were also observed in Switzerland (Hofer *et al.* 2000; Burlet *et al.* 2011) and Kyrgyzstan (Ziadinov *et al.* 2011).

The patchy distribution of *E. multilocularis* may reflect a variable distribution of favourable environments conducive to more intensive transmission. The distribution range of possible definitive hosts covers virtually the totality of the northern hemisphere, and susceptible intermediate host species (>40 to date) are virtually present in every possible terrestrial habitat of that area (Giraudoux *et al.* 2002; Vuitton *et al.* 2003). Temperature and relative humidity have an impact on tapeworm egg survival (Veit *et al.* 1995) and may be important limiting factors at various scales (Burlet *et al.* 2011). Moreover, correlations between land cover and spatial distribution of human AE have been evidenced in Eastern France, in South Gansu, in South Ningxia and on the Tibetan plateau of western Sichuan and Qinghai (Giraudoux *et al.* 2003, 2006, 2013; Wang *et al.* 2004, 2006; Pleydell *et al.* 2008). A similar correlation has been found for fox infection in France (Pleydell *et al.* 2004). Those correlations have been explained by the impact of land cover on intermediate host population dynamics. The amplitude of seasonal and inter-annual variations of population densities of some arvicolid and/or lagomorph small mammal species increases with the ratio of their optimal habitat to the total available habitat (Lidicker, 2000). This may lead to phases of large population densities where the biomass of the total population of intermediate host can reach several tens of kilograms per ha. During those periods, fox definitive hosts feed almost exclusively on those prey populations that are easy to access and are more likely to ingest infected small mammals even when prevalence is low (Raoul *et al.* 2010). These factors foster transmission (Giraudoux *et al.* 2002) and human exposure risk (Viel *et al.* 1999), the latter possibly via dogs feeding on abundant reservoirs of small mammal intermediate hosts close to towns, villages and rural settlements. However, even in areas where an overall higher prevalence of human AE was observed and the combination of apparent favourable climate and landscape was met, there still remained large unexplained differences between sampling units (generally villages) (Danson *et al.* 2003, 2004).

If landscape metrics could be used to predict areas of higher risk for *E. multilocularis* transmission, a key factor will be the presence of small mammal communities that are likely to include intermediate host species prone to reach large population densities within suitable habitats, in addition to climate variables that are likely to favour parasite egg survival (Giraudoux *et al.* 2013). Furthermore, within those areas predicted at higher risk, large unexplained differences between localities must be expected, due to the typical over-dispersed pattern of this helminth parasite. This can lead to discrete areas of 100–1000 square kilometres where human AE prevalence is more than 10–100 times larger than outside the high-risk zone. Those areas can be considered ‘hotspots’.

One particular feature of China and neighbouring countries of Central Asia, in contrast to Europe, is the extraordinary large variety of small mammal species and assemblages that covers this large region. Based on the comparison of mammal and plant species, a total of 25 biogeographical regions and 77 sub-regions have been defined in China (Xie *et al.* 2004), including more than 274 species of small mammals many of which are candidate intermediate hosts for *E. multilocularis* transmission. However, it has been shown that *E. multilocularis* transmission, although sustained in a large number of different small mammal communities, does not depend on intermediate host species richness, but rather on relatively low biodiverse communities in which some species are prone to significant population increase (Giraudoux *et al.* 2013).

In this article, we use our field experience and knowledge (over the past 20 years) about the ecology of different *E. multilocularis*/human AE transmission hotspots to attempt to define ecological conditions that characterize those optimal transmission ecosystems in China and neighbouring countries of Central Asia.

## HUMAN AE HOTSPOTS IN CHINA AND NEIGHBOURING COUNTRIES

**Fig. 1.**
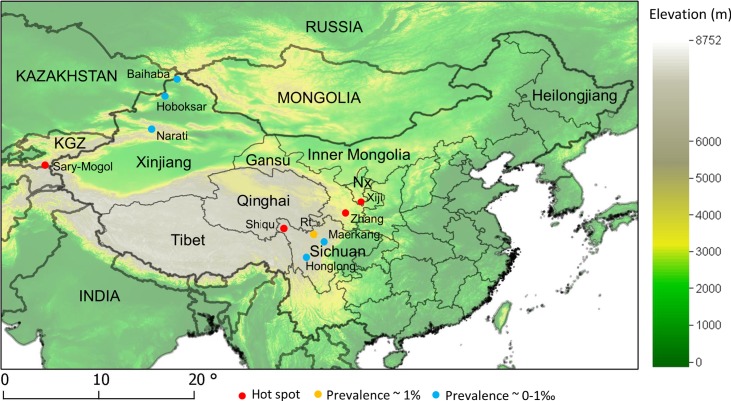
Map of China and main locations cited in the text. The background is the Global Land One-kilometre Base Elevation model, provided by the US National Oceanic and Atmospheric Administration. Coordinates reference system in degrees (WGS84).KGZ, Kyrgyzstan; NX, Ningxia; Rt, Rangtang.

In parallel to host ecology studies, mass screenings for human AE were carried out in known AE endemic sites (Fig. 1). Table 1 summarizes the main features of the transmission systems prevailing in each study site and three of them, Sary-Mogol (Kyrgyzstan), Shiqu (China) and Zhang (China) showed much greater human AE prevalence than the others. Five sites in western China (Narati, Hoboksar, Baihaba, Honglong and Maerkang) showed human AE prevalence about 10 times lower, while Xiji and Rangtang sites in China could be ranked intermediate. Clearly Sary-Mogol, Shiqu, Zhang and also Xiji were transmission hotspots, with human AE prevalence exceeding by as much as 10 times the average prevalence reported for the region as a whole. The whole range of those sites is actually included in the range of the main definitive host of *E. multilocularis*, the red fox, *Vulpes vulpes*. However, on the Tibetan plateau the parasite is likely to circulate mostly through the Tibetan sand fox, *V. ferrilata* (Jiang *et al.* 2012). Furthermore, the corsac fox, *V. corsac*, may be present in the drier areas of north China, and has also been found infected in Inner Mongolia and Kazahkstan (Xu *et al.* 1992; Bessonov, 1998; Shaikenov and Torgerson, 2002; Tang *et al.* 2004, 2006).

**Table 1.** Main characteristics of the areas studied. AE, number of human alveolar echinococcosis cases; n, number of people examined; in Sary-Mogol area (ae cases between parentheses and numbers in italics), only unpublished first estimations from ultrasound mass screenings carried out in 2012 by one of the co-authors (IZ) and co-workers are available: further investigations and PCR confirmations are yet currently in process

Location name	AE	*n*	Prevalence (95% CI between parentheses)	Landscape dominant habitat	altitude (m)	Dog population	Fox populations	Large populations of small mammals	Human AE	Ecology
Sary-Mogol	(*115*)	*1617*	*7·1* (*5·9, 8·5*)	Pamir alpine grassland (100%)	3000–3200	Large	*Vulpes vulpes* (large)	*Ellobius tancrei* *Microtus gregalis*	Ziadinov *et al*. (personal communication)	Giraudoux *et al*. (unpublished)
Shiqu	192	3196	6 (5·2, 6·9)	Eastern tibetan plateau grassland (100%)	4200–4500	Large	*Vulpes ferrilata* (large), *Vulpes vulpes* (confirmed)	*Ochotona curzoniae* *Microtus limnophilus* *Cricetulus kamensis*	Li *et al*. (2010)	Raoul *et al*. (2006), Wang *et al*. (2007, 2008)
Zhang	135	3331	4·1 (3·4, 4·8)	Shrubs, grassland (22–91%) and farmland (originally: sub-alpine mountain forest)	2300–2600	Varying	*Vulpes vulpes* (extremely low, if not extinct at the time of the study)	*Microtus limnophilus* *Cricetulus longicaudatus* *Eospalax fontanierii*	Craig *et al*. (2000)	Giraudoux *et al*. (1998)
Xiji	90	3629	2·5 (2, 3·1)	Farmland (90%) (originally: subalpine mountain forest)	2000–2200	Varying	*Vulpes vulpes* (likely extinct at the time of the study)	*Cricetulus longicaudatus* *Eospalax fontanierii*	Yang *et al*. (2006)	Raoul *et al*. (2008) and unpublished data
Rantang	10	675	1·5 (0·8, 2·8)	Mixture of subalpine mountain forest and grassland	3700–4000	Yes	*Vulpes vulpes*	–	Li *et al*. (2010)	Vaniscotte *et al*. (2009)
Narati	6	1838	0·3 (0·1, 0·7)	Mixture of subalpine mountain forest and grassland	1700–2000	Yes	*Vulpes vulpes*	*Ellobius tancrei* *Microtus obscurus*	Feng (2013)	Giraudoux *et al*. (unpublished)
Kokehada	2	1339	0·1 (0, 0·6)	Mountain grassland (100%)	1900–2100	Yes	*Vulpes vulpes*	–	Feng (2013)	Giraudoux *et al*. (2008*a, b*)
Baihaba	0	1644	0 (0, 0·3)	Mixture of subalpine moutain forest and grassland	1100–1300	Yes	*Vulpes vulpes*	*Ellobius tancrei* *Microtus obscurus*	Feng (2013)	Giraudoux *et al*. (2008*a, b*)
Honglong	0	610	0 (0, 0·8)	South-eastern tibetan plateau grassland (100%)	3950–4400	Yes	*Vulpes vulpes* (confirmed)	–	Li *et al*. (2010)	Giraudoux *et al*. (unpublished)
Maerkang	0	571	0 (0, 0·8)	Subalpine forest	2800–4100	Yes	*Vulpes vulpes*	–	Li *et al*. (2010)	Vaniscotte *et al*. (2009)

All these transmission hotspots share the fact that a number of small mammal intermediate host species were prone to population outbreaks during the study period, indicated by large trapping success and small mammal activity indices (Giraudoux *et al.* 1998; Raoul *et al.* 2006, 2008). Small mammal population surges were also found in Narati and Baihaba in Xinjiang (China), however they appeared not to translate into human AE prevalence levels as large as in the other sites highlighted above. Rangtang is situated less than some tens of kilometres from the high altitude grassland of the eastern Tibetan plateau where known population outbreaks occur for the plateau pika, *Ochotona curzoniae* and arvicolid species (e.g. Banma area, see Giraudoux *et al*. (2006)). It is probable that the small mammal survey we conducted in this area was carried out during a transitory low density phase of small mammal population fluctuation (Vaniscotte *et al.* 2009). However, Rangtang is in the vicinity of a larger scale region where small mammal population densities were higher, and the exposure of Tibetan nomads travelling on the plateau may explain the unexpectedly large human AE prevalence we observed there.

**Fig. 2.**
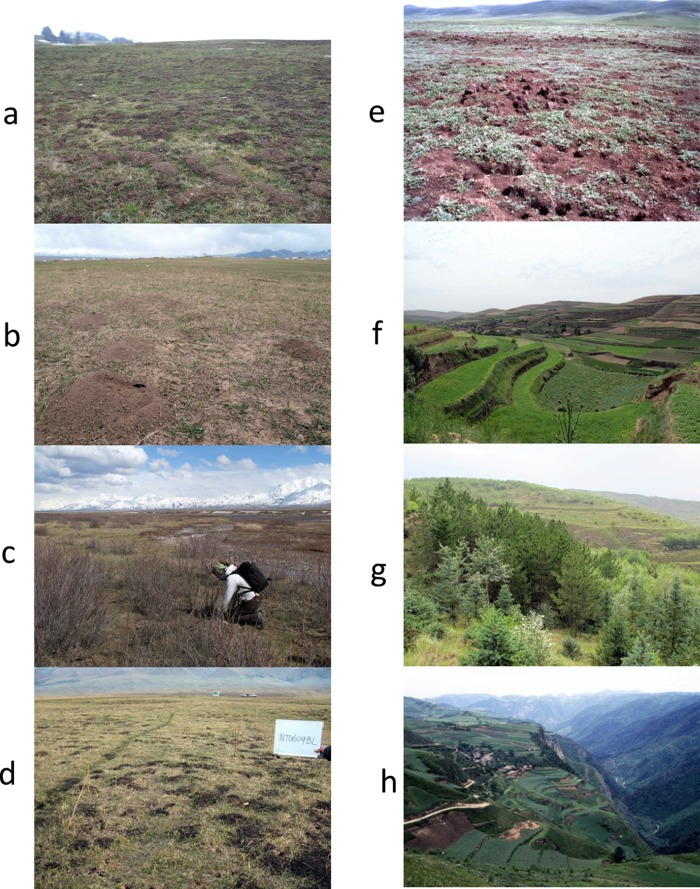
View of some of the hotspots studied. a, Outbreak of *Arvicola terrestris* and *Microtus arvalis* in the Jura massif grassland, France (*tumuli* are earth expelled by *A. terrestri*s when digging galleries), b, outbreak of *Ellobius tancrei*, Sary-Mogol grassland, south Kyrgyzstan (note the typical conical *tumuli* with lateral opening); c, typical habitat of *Microtus gregalis* along streams, Sary-Mogol, south Kyrgyzstan; d, outbreak of *Ellobius tancrei* and *Microtus obscurus*, Narati, Tien Shan, Xingjiang, China; e, outbreak of *Ochotona curzoniae*, Tuanji, eastern Tibetan plateau, Sichuan, China; f, field terraces, Xiji, Ningxia; g, grassland and afforested areas in Xiji. Field terraces and early stages of recently afforested areas are typical habitats for *Eospalax fontanierii* and *Cricetulus longicaudatus*; h, in 1996, field terraces of Ban Ban Wan (human AE prevalence: 16%) and deforestation in progress on the other side of the valley, Zhang, Gansu, China.

Clearly the human AE disease hotspots reported in this review represent transmission ecology characteristics favourable to population surges of some small mammal reservoirs (Fig. 2). In Shiqu (eastern Tibetan plateau, Sichuan, China) and Sary-Mogol (southern Kyrgyzstan) transmission landcapes were characterized by alpine grassland (Giraudoux *et al.* 2013 and unpublished), while in Zhang (south Gansu, China), transmission was occurring in a mixed landscape of sub-alpine shrub, grassland and farmland resulting from massive deforestation (with greater AE prevalence in villages with larger ratio of shrub and grassland) (Giraudoux *et al.* 2003). In Xiji (south Ningxia, China), the landscape was largely dominated by terraced farmland and grassland developed after clearance of sub-alpine forests whose degraded remnants could be found only on the top of local hills (Pleydell *et al.* 2008; Yang *et al.* 2012). In all of those landscapes, the permanent (e.g. Tibetan plateau) or transitory (e.g. during deforestation processes in Zhang and Xiji) extension of habitats favourable to cyclic species of small mammals was a key factor for *E. multilocularis* transmission. This fits quite well with observations made in western Europe, where arvicolid population outbreaks and larger prevalence of *E. multilocularis* in red fox populations and of human AE, correlated with the extension of permanent grassland in mid-altitude mountains (Viel *et al.* 1999; Giraudoux *et al.* 2003; Pleydell *et al.* 2004). A continental-wide increase of red fox population density after rabies vaccination campaigns has further complicated this pattern (Chautan *et al.* 2000; Schweiger *et al.* 2007) increasing *E. multilocularis* prevalence in the known endemic areas and extending areas of large prevalence to new areas with no small mammal population outbreaks.

In China and some neighbouring central Asian countries, endemic landscapes are associated with different small mammal communities belonging to various biogeographic areas (Giraudoux *et al.* 2006, 2013). On this basis, we hypothesise that transmission hotspots of *E. multilocularis* may occur in favourable climatic areas where large areas of grasslands (e.g. alpine, sub-alpine or plain) can be found and that meet the distribution range of rodent and/or ochotonid species prone to population surges. We believe this assumption can serve to establish risk maps on a continental scale and guide further research on human AE distribution at finer spatial resolution. It may also help to define transmission ecosystems using some species of small mammal as flagship or indicator species that are species signalling ecological conditions by which transmission of *E. multilocularis* may be sustained more intensively.

## TOWARDS PREDICTING AT RISK AREAS AT CONTINENTAL SCALES

### Data and methods

Keystone, flagship and umbrella species are conservation buzzwords to serve as shortcuts or proxies for conserving background biodiversity (Barua, 2011). In conservation, flagship species are ‘popular charismatic species that serve as symbols and rallying points to stimulate conservation awareness and action’ (Heywood, 1995). Here we propose to apply this concept to parasite ecology, as small mammal host species that can serve to signal ecosystems in which *E. multilocularis* transmission can be especially fostered, and promote them to public health awareness. We do not mean that a flagship species is necessarily the main route for transmission, although it can often be the case (it would therefore also be a keystone species for transmission). In our case, ideally, a flagship species should be easy to detect and to identify, linked as much as possible with a specific ecosystem that has a high transmission potential (due to the presence of this species or due to the fact that other intermediate host species with a high transmission potential are associated with this species). Furthermore, their distribution area should not or only marginally overlap on a regional scale with other flagship species. Here, species selected as flagship species were either those shown to be subject to population outbreaks in our studies (Table 1) or from the literature (Tang *et al.* 2004; Winters *et al.* 2010), easier to identify and typical of the regional small mammal assemblages. Data on small mammal and carnivore species range were provided by the International Union for Conservation of Nature (IUCN): http://www.iucnredlist.org and were updated based on Giraudoux *et al*. (2008*b*) in China and our own field observations in Kyrgyzstan. Species nomenclature was standardized according to Wilson and Reeder (2005), except for *Microtus obscurus*, the Altai vole, that has been recently shown to be different from *M. arvalis*, the common vole (Tougard *et al.* 2013).

Very few studies (and none in Asia) provide quantitative information on climatic conditions that may limit *E. multilocularis* distribution. Miterpakova *et al*. (2006), in Slovakia, have shown that *E. multilocularis* distribution in foxes was limited by moderate to higher annual air temperature, lower annual rainfall and soil drought. Parasite prevalence was clearly much larger in areas with average annual temperatures lower than 9–12 °C. No evidence of *E. multilocularis* transmission in France was found where the average annual temperature was higher than 12 °C (Giraudoux, unpublished, derived from the 0·5 degree gridded dataset of the FAO Global maps (Leemans and Cramer, 1991), http://www.fao.org/sd/EIdirect/climate/EIsp0002.htm). Here, in the current study in China and neighbouring countries of Central Asia, we assumed, for lack of better proxy to climatic conditions, that *E. multilocularis* egg survival was unlikely if average annual temperature was higher than 12 °C.

Information on land cover in China and neighbouring countries was obtained from the Global Land Cover 2000 project http://bioval.jrc.ec.europa.eu/products/glc2000/products.php. It was based on Spot Vegetation data from the period 1 January to 31 December 2000, and consisted of 31 land-cover classes mapped at a pixel resolution of 1 km. Here category 7 corresponds to the relatively rich grassland of the alpine and sub-alpine vegetation belt described above (see also Giraudoux *et al.* (2013)). To take account of the possible effects of large areas of grassland in the surroundings of a given locations, a neighbourhood analysis was carried out. For every pixel of the map, we computed the ratio of grassland to the total area within a radius of 31 km.

## RESULTS

**Fig. 3.**
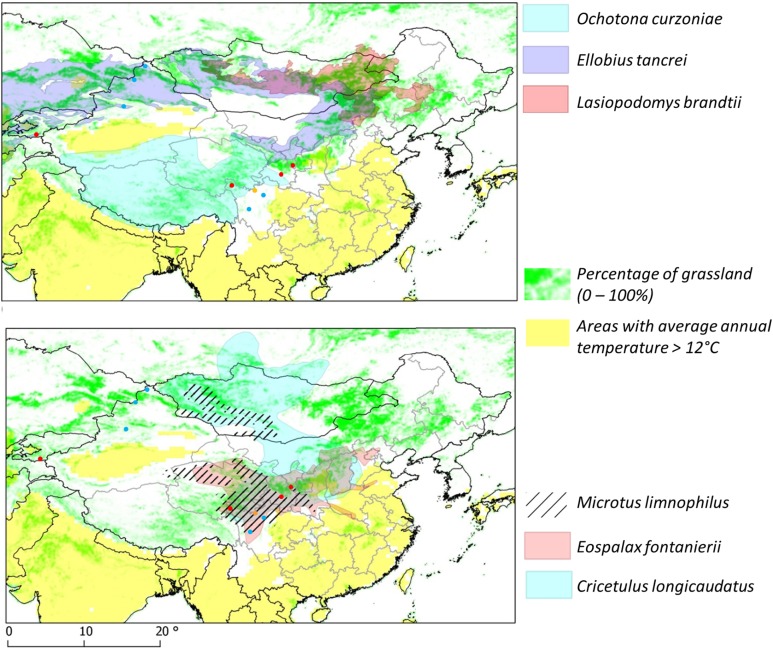
Grassland land cover and distribution range of flagship species, after IUCN (2012), modified.

The hotspots described above were all located in areas with an actual or potential larger percentage of grassland (Giraudoux *et al.* 1998, 2003, 2013; Raoul *et al.* 2006; Vaniscotte *et al.* 2009). Furthermore, four major communities of small mammals can be identified in those areas which characterize a transmission system (Fig. 3).

### Eastern Tibetan plateau

Grassland small mammal communities are characterized by the presence of *O. curzoniae* (plateau pika), selected here as the flagship species, because being diurnal it is easily detected and easy to identify, directly or based on its indices (holes, faeces, etc.). Furthermore its distribution range does not overlap other transmission systems. Its geographical distribution is grossly the same as that of *Phaiomys leucurus* (Blyth's mountain vole, formerly *Microtus leucurus*). On the eastern border of the plateau, some other species such as *Microtus limnophilus* (lacustrine vole), *Lasiopodomys fuscus* (smokey vole, formerly *Microtus fuscus*), *Neodon irene* (Irene's moutain vole, formerly *Microtus irene*), *Cricetulus kamensis* (Tibetan dwarf hamster) and *Ochotona cansus* (Gansu pika), can also locally reach large population densities (Raoul *et al.* 2006) and contribute to transmission. All species except *O. cansus* have been found naturally infected with *E. multilocularis*. *L. fuscus* is confusingly still cited maybe erroneously in parasitology literature. Its actual distribution range appears to be limited to south Qinghai, while most citations likely concern *P. leucurus*, a widespread species quite similar morphologically. In fact, the two species were pooled into a single species in the past (Qiu *et al.* 1999; Raoul *et al.* 2006). Definitive hosts in the wildlife cycle are typically the Tibetan sand fox (*V. ferrilata*) and the red fox (*V. vulpes*) and both proved naturally infected (Jiang *et al.* 2012). Dogs are also commonly infected in those areas (Budke *et al.* 2005*a*, *b*; Vaniscotte *et al.* 2011). Observations of dog preying on small mammals in and around human settlements are common and small mammal consumption evidenced from dietary analysis (Wang *et al.* 2010). However, the details of the parasite transmission routes in this area are still far from complete. For instance, we do not know the relative contribution of the various intermediate host species to the parasite life-cycle. Possible inter-annual small mammal population density variations in both space and time may provide a meta-stable transmission system involving fox as well as dog definitive hosts (Giraudoux *et al.* 2006). However, the respective contribution of dog and fox in the cycle is still unclear locally. One may suspect that in some areas, most of the parasite population biomass is in the dog population in communities (Budke *et al.* 2005*a*). On the Tibetan plateau or elsewhere for that matter, evidence of a dog population infected with no link to a sylvatic cycle (involving foxes) is lacking, which poses the question of transmission sustainability in an isolated population of dogs. However, a recent dog reinfection study in a Tibetan area of Sichuan suggests that dog populations are reinfected quickly and may contribute substantially to an active peri-domestic cycle (Moss *et al*. – see this Special Issue of Parasitology Vol. 140, 2013).

### Altai, Tien Shan and Pamir

**Fig. 4.**
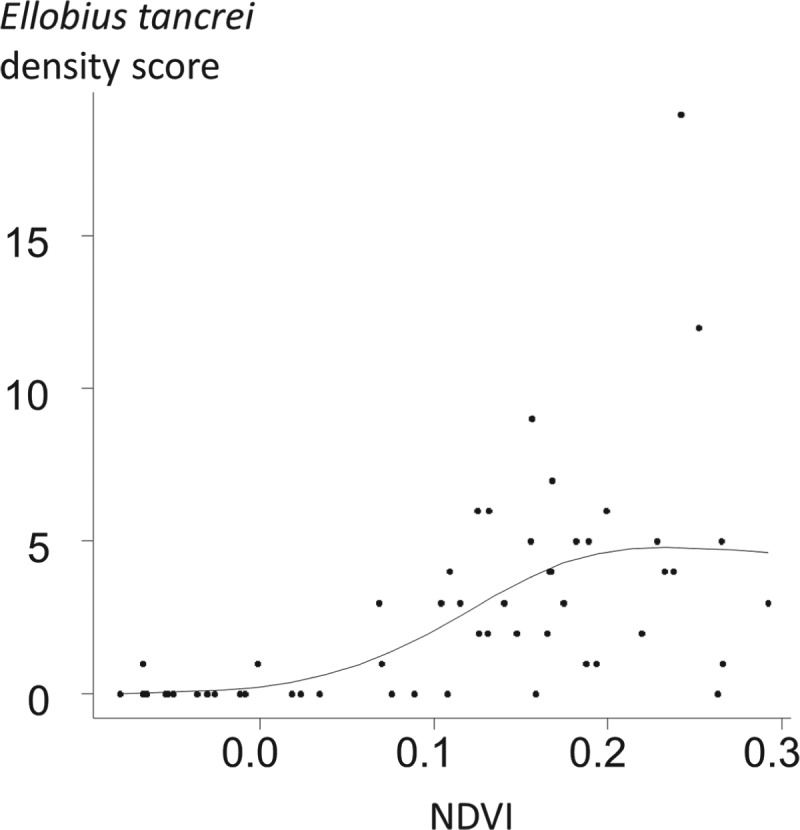
Relative density of *Ellobius tancrei* population and grass density in Sary-Mogol, Alay Valley. 56 locations were selected at an average distance of 1·2 km from each other. 20 intervals of 10 paces were walked at each point in may 2012. The score of relative density of *E. tancrei* was the number of intervals where fresh *E. tancrei* indices could be observed (see e.g. Giraudoux *et al*. 1995; Quéré *et al*. 2000). The NDVI (Normalized Difference Vegetation Index), an index commonly used to estimate the relative density of plant cover, was computed from a Landsat ETM image acquired the 16th of June 2004. The relationship between *E. tancrei* score and NDVI was modelled using a general additive model with a Poisson error, and a thin plate regression smoother (using a three dimensional basis) (Wood, 2006). The empirical variogram of residuals was included within the limits of the variogram envelop obtained from 99 random permutations of the original data, indicating that no spatial autocorrelation could be detected. The correlation between *E. tancrei* score and NDVI was found statistically significant at a probability lower than 0·00001.

**Fig. 5.**
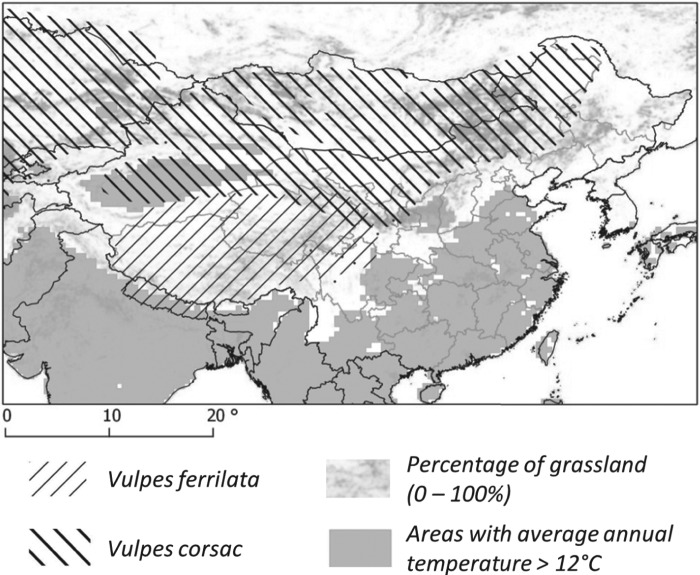
Distribution of fox species. The distribution of the red fox covers the totality of the area.

*Ellobius tancrei* (eastern mole vole) is a flagship species for small mammal communities and for an *E. multilocularis* transmission ecosystem, whose range stretches from the Pamir mountains (south Kyrgystan), through southern Kazakhstan to northern Xinjiang (Tien Shan and Altai mountains). It probably also includes western and central Mongolia where it meets the eastern small mammal assemblage (see below). This species is typical and easy to identify, directly or based on its cone-shaped earth tumuli with a middle hole from which a breach is maintained on one side. The population density of *E. tancrei* increases with grassland productivity (Fig. 4). It has been found naturally infected in Kyrgyzstan (Tokobaev, 1959; Afonso *et al*. unpublished). Additional potentially outbreaks of arvicolid species can be found more or less locally; for example, *Microtus gregalis* (narrow-headed vole), *M. oeconomus* (root vole) along streams and in marshes and *M. obscurus* (Altai vole), in grassland (Giraudoux *et al.* 2008*b*; unpublished). The red fox is the typical definitive host in the wildlife cycle, and domestic dogs in villages, hamlets and isolated farms of the Alay valley (Kyrgyzstan) and in Mongol and Kazak settlements of northern Xinjiang. The contribution of the corsac fox (*V. corsac*) can be questioned locally (Fig. 5), but this species is generally not observed in mountainous areas or in regions where snow depth exceeds 150 mm. On this basis, it is not expected to be present in the alpine and sub-alpine grassland of the mountain ranges of this part of the world (Smith and Xie, 2008; Wilson and Mittermeier, 2009).

### Eastern Mongolia and Inner Mongolia

*Lasiopodomys brandtii* (Brandt's vole, formerly *Microtus brandtii*) characterizes transmission systems of eastern Mongolia and eastern Inner Mongolia and has been found naturally infected (Tang *et al.* 2004). It can reach large population densities and cause severe damage to grassland (Winters *et al.* 2010). The role and the importance of the Daurian pika (*Ochotona dauurica*), in this area are uncertain. This species is sometimes cited as a potential grassland pest in the literature (Komonen *et al.* 2003) and has been found naturally infected in the Tuva Republic, Russia (Vuitton *et al.* 2003). Both the red fox and the corsac fox are present. Tang *et al*. (2004) provide evidence of *E. multilocularis* infection for the latter species, as well as for *L. brandtii* and *Meriones unguiculatus* (Mongolian jird).

### South Gansu and south Ningxia

These provinces/autonomous regions of central-west China share very similar communities thriving in a mosaic of grassland and farmland originating from massive deforestation of sub-alpine forests until the early 1990s. In farmland and newly reforested areas they are dominated (in terms of biomass) by *Cricetulus longicaudatus* (long-tailed dwarf hamster) and *Eospalax fontanierii* (Chinese zokor) (Giraudoux *et al.* 1998; Raoul *et al.* 2008), the latter species being selected here as flagship species. This species is easy to identify and well known by farmers who trap them actively in spring for crop protection. It leaves unmistakable indices such has holes of 10 cm diameter in fields and fallows, often closed by an earth cap. In the western part of the area (e.g. Gansu), *M. limnophilus* can also reach large population densities in shrubby and grassland areas. In southern Ningxia, we did not find evidence of arvicolid populations, except in the Liu Pan Mountains (Raoul *et al.* 2008; Giraudoux *et al*. personal observation in 2012). This suggests that south Ningxia may so far be the only place in the world, where *E. multilocularis* transmission (with resultant high human incidence) occurs without an arvicolid species being involved. The corsac fox is known to avoid cultivated land and although south Ningxia is included in the theoretical distribution range of *V. corsac*, one can assume the fox is absent in the human AE hotspots. Furthermore red fox populations, although present and still locally abundant in the 1980s, have considerably decreased and are virtually absent in areas of intensive farming such as in the human AE foci around Xiji (Yang *et al.* 2012, local farmer interviews, local CDC reports and Giraudoux *et al*. personal observation). Subsequently, the only possibility for the parasite to be maintained would be to circulate through a dog/zokor and/or a dog/hamster lifecycle (possibly involving locally a range of other species such as *Spermophilus dauricus*, the Daurian ground squirrel, and possibly some species of the Cricetinae, Gerbillinae, Dipodidae and Ochotonidae) (Raoul *et al.* 2008; Yang *et al.* 2012). As everywhere else in the world, dogs may themselves catch and eat small mammals. During a 2012 field survey in south Ningxia, ground squirrel populations had dramatically crashed and just one specimen could be observed whereas hundreds were visible in 2003 (Giraudoux *et al.* personal observation). In the early 1990s in Ningxia, people reportedly used to trap ground squirrels (*Spermophilus* spp.) for family meat consumption and questionnaires to human AE patients from south Ningxia indicated that viscera with multiple macro-cystic lesions on the liver were used to feed dogs (Li *et al.* 1990; Wang *et al.* 1991). Furthermore, in spring, zokors (*E. fontanierii*) are usually caught in large numbers by farmers for crop protection and are also often given as food to dogs (local villagers and local CDC staff, personal communication). Moreover, Pleydell *et al.* (2008) found that drinking water not originating from tap or a well was a risk factor for human AE. This suggests that water possibly contaminated by dog faeces might be an additional source of human infection. Actually the general pattern for sustainable transmission of the parasite in south Ningxia is still more an enigma than in the other areas studied in China. South Ningxia may be the only place in the world yet described where *E. multilocularis* is maintained through a small mammal/dog cycle that is not connected to a sylvatic cycle involving foxes, but clear evidence of this is required.

**Fig. 6.**
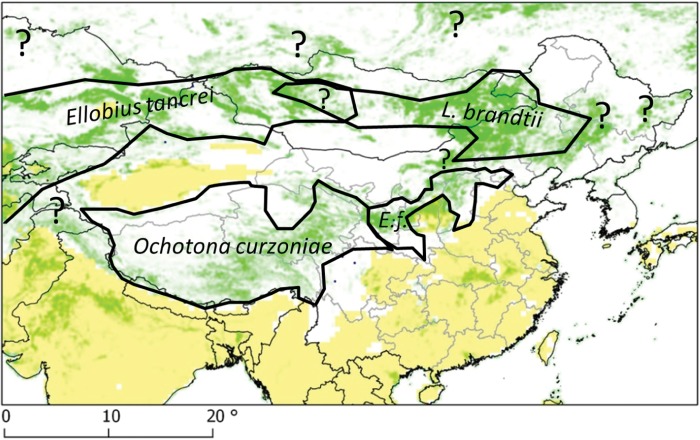
Transmission systems and their flagship species. Except in the *Eospalax fontanierii* area (*E.f.*), more intense transmission and hotspots are more likely found in areas with larger ratio of permanent grassland. Question marks are placed on a number of areas of large ratio of permanent grassland but where no reference studies on small mammal communities and transmission are yet available.

Based on our knowledge gained in a number of *E. multilocularis* transmission hotspots studied in China and neighbouring countries of Central Asia, the aim of this review is to identify and describe the general characteristics of transmission ecosystems in that Region. The large complexity that may arise from the presence of hundreds of potential candidate small mammal (intermediate host) species can be overcome by consideration of a few flagship species of small mammal intermediate hosts that are prone to population surges and are embedded within suitable landscapes with an optimal combination of climate and land cover characteristics (Giraudoux *et al.* 2013). Using this approach, we have been able to identify 4 types of small mammal assemblages each characterized by a different flagship species; i.e. *O. curzoniae, E. tancrei, L. brandtii* and *E. fontanierii* (Fig. 6). These 4 small mammal species are all adapted to the cold climates typical of alpine and sub-alpine grasslands. In first three of them, small mammal population density is dependent on the response of grassland species to the availability and productivity of their grassland habitat within the given landscape. The fourth species, *E. fontanierii*, occurs in areas of south Ningxia (central China) which were originally mapped as sub-alpine forest or grassland. Those habitats have now been transformed into farmland. In such expanded agricultural areas, the only small mammal species that reaches large population biomass over large scales are zokors and hamsters, and these hosts probably indicate a unique transmission ecosystem within farmland where no arvicolid rodents appear to participate.

Of course, this descriptive assessment is likely to be an oversimplification of reality. For instance, our knowledge of the details of the distribution range and ecology of small mammal host species is still very poor for much of central and eastern Eurasia (most IUCN maps available have been corrected based on data collected in our field surveys for this article). Furthermore, the concept of ‘flagship small mammal species’ and their association with a putative transmission system that we have proposed here must not hide the possible role of other key intermediate host species present in the target ecosystem and that could be involved in the sustainable transmission of *E. multilocularis*. In addition, undetected intermediate host species may occur whose detailed distributions will be poorly known or even unknown. For example, no description of an *E. multilocularis* transmission ecosystem has yet been proposed for the known areas of endemicity located in the southern part of Russia bordering China and Mongolia. In north-east China, for example, no single candidate flagship small mammal species can be proposed so far for the transmission system(s) responsible for local cases of human AE in Heilongjiang (Zhou, 2001; Danson *et al.* 2003). There also remains much uncertainty about the general characteristics of transmission ecosystems where they overlap, e.g. between the Kunlun and Pamir mountains or in central Mongolia (Fig. 6).

Furthermore, large sciurid rodents such as *Marmota* spp., *Spermophilus* spp. and large lagomorphs, such as hares, are present in mountain grasslands and their populations locally may be relatively large. Because these mammals are relatively long lived intermediate hosts they may act as long-term reservoir hosts for *E. multilocularis* (Zhou *et al.* 1998, 2000; Xiao *et al.* 2004; Yang *et al.* 2012), compared to the rapid turnover of a vole population which is almost totally renewed each year. Another enigma is the possible role in sustainable transmission of this parasite in semi-desert small mammal species e.g. gerbils (*Meriones* spp.) jerboas (*Allactaga* spp. and *Dipus* spp.), hamsters, (*Cricetulus* spp.), and other candidate mammals. These small mammal communities of arid environments tend to stay at much lower population densities due to the low primary production of the ecosystem they inhabit and to periods of long seasonal drought (the latter unfavourable to parasite egg survival). However, these arid ecosystem rodent communities can possibly meet and partly overlap with other small mammal communities at the edge of their ecological distribution (e.g. along streams, see Shaikenov and Torgerson (2002) and Tang *et al.* (2004)). We do not know whether those arid-adapted small mammal populations can sustain the transmission of *E. multilocularis* by themselves in the long term or are only sporadically exposed from parasite spill-over, for example via fox movements, from neighbouring communities (e.g. arvicolids) or from ecosystems that are more favourable for intensive transmission.

## CONCLUSIONS

A striking feature of the transmission ecology of *E. multilocularis* in China and in some neighbouring countries of Central Asia is the diversity of potential small mammal intermediate hosts and the range of ecological and anthropogenic drivers (e.g. climate, geography, grassland management, deforestation, reforestation) that directly or indirectly contribute to reinforce or limit the parasite lifecycle in sylvatic mammals. The distribution of small mammal assemblages, their ecological relationships, and even their basic taxonomy in China and Central Asia are far from completely understood, but the emergence of reasonably comprehensive atlases in term of species number and distribution have been published recently (Smith and Xie, 2008; IUCN, 2012). Nevertheless, the lack of information about small mammal populations may be a considerable obstacle for understanding transmission patterns of *E. multilocularis* at relevant spatial scales, and thus for subsequent public health awareness and zoonosis prevention options.

Based on the knowledge acquired in areas of China and Central Asia, where both small mammal host ecology and the epidemiology of human AE were studied in parallel, we strongly recommend an ecological approach that defines large spatial units or transmission ecosystems of *E. multilocularis* wherein general and specific environmental features may indicate or even predict ‘risky’ or ‘unhealthy’ landscapes (Patz *et al.* 2004). Those units or ecoscapes (Lidicker, 2008) can be characterized by flagship small mammal host species whose distribution corresponds to ecological conditions more likely to drive intensive transmission. This may happen when landscape characteristics and climatic conditions combine together with the extension of optimal habitats for some small mammal species prone to population outbreaks. The relative role of each of those particular small mammal species in transmission of *E. multilocularis* can hardly be quantified at this stage, not least because of the extreme complexity of their multi-annual population dynamics with a cascade of consequences on the host prey/predator relationships and on parasite transmission (Giraudoux *et al.* 2007; Raoul *et al.* 2010). Nevertheless, taken as whole, each putative transmission ecosystem with its own biotic and abiotic characteristics can serve as the basis for further in- depth research and in the long term help decision makers to implement surveillance systems and prevention for this pathogenic zoonosis.
